# An LSTM-Method-Based Availability Prediction for Optimized Offloading in Mobile Edges

**DOI:** 10.3390/s19204467

**Published:** 2019-10-15

**Authors:** Chaoxiong Cui, Ming Zhao, Kelvin Wong

**Affiliations:** 1 School of Computer Science and Engineering, Central South University, Changsha 410000, China; cuichaoxiong@csu.edu.cn; 2 School of Electrical and Electronic Engineering, University of Adelaide, Adelaide, SA 5005, Australia; kelvin.wong@adelaide.edu.au

**Keywords:** mobile edge computing, cognitive computing, deep learning, computation offloading, mobility, optimization

## Abstract

Mobile edge computing (MEC) can augment the computation capabilities of a vehicle terminal (VT) through offloading the computational tasks from the VT to the mobile edge computing-enabled base station (MEC-BS) covering them. However, due to the limited mobility of the vehicle and the capacity of the MEC-BS, the connection between the vehicle and the MEC-BS may be intermittent. If we can expect the availability of MEC-BS through cognitive computing, we can significantly improve the performance in a mobile environment. Based on this idea, we propose a offloading optimization algorithm based on availability prediction. We examine the admission control decision of MEC-BS and the mobility problem, in which we improve the accuracy of availability prediction based on Empirical Mode Decomposition(EMD) and LSTM in deep learning. Firstly, we calculate the availability of MEC, completion time, and energy consumption together to minimize the overall cost. Then, we use a game method to obtain the optimal offloading decision. Finally, the experimental results show that the algorithm can save energy and shorten the completion time more effectively than other existing algorithms in the mobile environment.

## 1. Introduction

Mobile and Internet of Things(IOT) devices have become an integral part of our daily lives [1,2]. With the emergence of emerging services, computing demanding applications touch every aspect of human activity. A typical mobile scenario is the Internet of Vehicles. A series of highly computationally intensive and complex services such as augmented reality, parking positioning, emergency situations on congested roads [3]. A large number of devices are used to generate more and more data. Those services require higher computing power. However, VT has limited computing capacity due to the limitation of physical size, which poses a major challenge to these computationally resource-intensive services [4,5].

In order to handle a large number of complex and intensive tasks, cloud computing has been proposed for storage and data processing [6,7]. While cloud computing is a centralized pool for configurable and powerful computing resources, its network latency is often unpredictable. The rapid growing distributed data further make it impractical to transfer all data to a remote cloud [8].

MEC has recently become a new computing paradigm that enables field data to be processed on network edges, mobile devices and connected devices [9,10]. It integrates IT service environment and cloud computing functions. MEC mainly implements computationally intensive and delay-critical applications, which greatly reduces delay and mobile energy consumption.

The Cellular Base Station (BS), which is considered to be a key driver of the MEC, has a storage function similar to cloud computing, serving the end user’s computing request. By adding a MEC server to the base station, the traditional base station becomes a MEC-BS, which enhances the VT’s ability from heavy computing resource load while reducing latency and avoiding congestion. In other words, MEC-BS can help VT to handle services, speed up processes, reduce service energy consumption.

Because of the exponential growth of computing services, MEC-BS cannot provide unlimited computing offload services for VT. If too many tasks offloaded from the VT, it will lead to MEC-BS queuing, delay or overload. In the existing work, these schemes attempt to reduce the load on the MEC-BS by rejecting, deferring or queuing the unloading request of the VT. At this time, the connection may be intermittent, resulting in service interruption. Therefore, it is especially important to design a new cognitive computing architecture to effectively manage MEC-BS resources.

In order to achieve effective management of the MEC-BS, it is important to consider the following questions: (1) How to make predictions? (2) Which tasks are offloaded to MEC-BS? (3) How to minimize the overall cost (completing time, energy consumption and availability of MEC-BS)?

Aiming to solve the above problems, we propose an optimized offloading method by predicting availability based on deep learning. The main contributions are as follows:

1. By predicting the availability of MEC-BS, we can reserve resources for tasks in advance, thereby reducing the heavy load of MEC-BS and ensuring service connectivity. The availability of MEC-BS is related to two aspects. The first is the admission control of the MEC-BS, and the other is the user’s dwell time, and then we decompose it into several prediction problems based on EMD, then separately predict based on LSTM method, and finally use the sum of predicted sub-data results as the output of the entire model.
2. In our optimization algorithm, local computing and MEC-BS computations were considered separately. They evaluated energy efficiency costs (EEC)(completing time, energy consumption and availability of MEC-BS), then transforms our algorithm into EEC minimization. The weight of the above three aspects is used to adjust the deviation between them, and we can flexibly adapt to different needs.
3. In order to solve the optimization problem, we use game theory to solve this problem. The above problem is defined as a distributed potential game problem by studying the limited improved properties and the potential game Nash Equilibrium.

The rest of this paper is organized as follows. The second section reviews related work, the third section introduces the system model and the proposed algorithm. The fourth section solves the proposed algorithm through the game, and the fifth section describes how to predict the availability of MEC-BS. The sixth section gives the experiment and evaluation of the algorithm, and the seventh section is a summary and future expectation.

## 2. Related Works

Computational offloading has always been a core issue in mobile edge/fog/cloud computing research [9]. The main problems are designing performance, energy consumption, intermittent connection, etc. In recent years, some research results have been achieved in this field, and we have summarized these studies as follows:

### 2.1. Computational Offloading Performance and Energy Consumption Issues

The main problem involved in this part is how to improve the efficiency of uninstallation and how to allocate resources reasonably. Kaddi et al. [11] presented a new energy-efficient clustering protocol that uses objective functions and random search jumps to reduce sensor energy consumption. Yang et al. [12] pointed out the minimized energy consumption problem of objective function combined with uplink and downlink. Guo [13] showed the problem of joint offloading and resource allocation based on energy consumption and completion time constraints, and then used the game model to get the optimal solution. Du [14] also studied joint transmission power, wireless bandwidth, and computing resources while ensuring user fairness and maximum tolerable delay. Chen and Zhang [15,16] proposed multi-user MEC computing offloading problem in multi-channel wireless environment, and designed distributed game theory offloading scheme. Chen [17] employed a method of offloading to a mobile cloud network by predicting bandwidth and computing rate to minimize resource consumption and meet delay requirements.Deng [18] proposed a workload dynamic scheduling algorithm, which can maximize the average throughput utility while guaranteeing the task processing delay in the worst case. The above papers only consider the computational offloading of performance problems, since the efficiency will be greatly reduced in case of connection interruption problems.

### 2.2. Computational Offloading Problem in the Internet of Vehicles Scenario

In recent years, the Internet of Vehicles has received extensive attention as a specific application scenario. Hou [19] pointed the vehicle fog computation (VFC) architecture, using near-user equipment and vehicle cooperation for communication and computation. Yu [20] provided a game model for management and contribution computing resources between different service providers. Mahadev [21] proposed a flexible offload strategy to perform task migration by combining in-vehicle cloud with infrastructure-based cloud. Liu et al. [22] raised the computational offloading problem in multi-vehicle edge networks, expressing the problem as a game problem of multi-user computing offloading. Zhang [23] considered the computational transfer strategy of vehicle-to-infrastructure (V2I) and vehicle-to-vehicle (V2V) communication modes greatly reduces the delays in computational offloading and transmission costs. Wang et al. [24] proposed a novel spider-web-like transmission mechanism for emergency data (TMED) in vehicular ad hoc networks, which improved the packet delivery ratio and average transmission delay of emergency data. Due to the need to consider the mobility environment, the Internet of vehicles is selected as a suitable scenario in this paper. However, this paper focuses on the architecture of Internet of vehicles, which is not considered together with performance in the target problem.

### 2.3. Intermittent Connection Problems in Mobile Edge Network Architecture

Most of the above studies have relatively stable edge computing connection systems. However, as described by Klein [25], since the user is mobile, there may be intermittent connectivity issues between the smart device and the network environment.The key to distinguishing between traditional cloud and mobile cloud systems is whether they can be persistently connected. The traditional cloud request method is intermittent connection on demand, and the control signal requirements in the mobile cloud system are more durable. In the current popular network systems, The presence of an intermittent connection may cause the offload request of the user to fail, which forces the user to request again. Almeida and Hoang [26,27] claimed intermittent connections caused by cloudlets. For example, due to limited resources, the cloud can reject a user’s offload request.In particular, the authors [26,27] believe that the revenue and QoS requirements of cloud providers are calculated based on the acceptance control and resource allocation of mobile users. [26] optimized the problem by a queuing-based approach. [27] redefine the problem as a semi-Markov decision process by introducing admission control and resource allocation. They all only considered the ideal situation (i.e., did not consider the persistent connection problem). The current and future connection modes are unknown when the user is moving.

In summary, the algorithm schemes proposed in the above work in performance, internet of vehicles scenario and mobile edge network architecture can effectively solve the problem of computational offloading under different constraints and scenarios. However, when both mobile environments and MEC-BS overloads are satisfied, none of the MEC-BS load mitigation issues are considered. we propose the computational offloading method based on Deep Learning. In the user’s movement process, we obtain the acceptance probability and dwell time of a series of vehicle tasks, so as to predict the availability of MEC-BS at the next moment. The obtained objective function formulated the optimal offloading strategy for the vehicle through game theory.

## 3. System Model and Formulation

### 3.1. Network Model

**Assumption** **1.**

*1.*  *The mutual interference between the vehicle and the vehicle, MEC-BS and MEC-BS are negligible.*
*2.*  *A vehicle can request one of MEC-BS servers for request task processing.*
*3.*  *The vehicle trajectories follow the poisson distribution [23], all vehicles travel in the same direction at a constant speed.*

**Figure 1:**
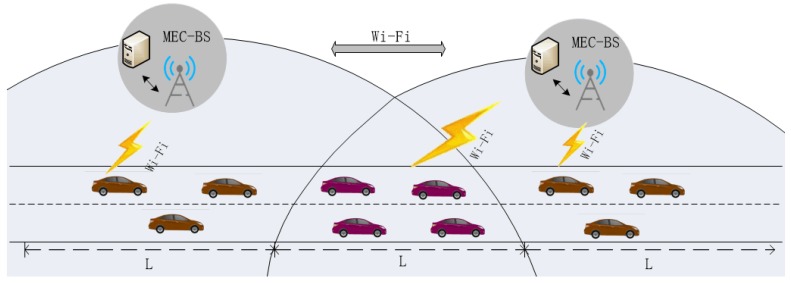
Network Architecture.

The mobile edge vehicle network architecture as shown in Figure 1, we consider a scenario consisting of MEC-BS. They can provide offload services.

There are a series of vehicles, each vehicle has a number of computing tasks, each of which can be optionally executed locally or offloaded to MEC-BS.

There are a series of computing tasks M expressed as $<L_{mv},D_{mv},S_{mv},t_{max}>$, which is characterized by: (1) $L_{mv}$, the amount of computing resources required to complete task M, for example, it can be quantified to the number of central processing unit cycles required; (2) $D_{mv}$, the size of the input file for some information about the task, such as program code or recorded video; (3) $S_{mv}$, the size of the received result, which is ignored here. It is much smaller than the transmission time; (4) $t_{max}$ is the maximum tolerance time for the task.

For convenience, the main symbols used in this paper and their specific meanings are summarized in Table 1.

### 3.2. Communication Model

We assume that the offloading decision of task m of vehicle v(MV) is: $A=a_{mv}|m\in M,v\in V$, and $a_{mv}=0$ means that MV is executed locally. $a_{mv}=1$ means that MV is offloaded to the MEC-BS. The uplink data rate for computation offloading of MV is:

(1) $$R_{mv}=Wlog_{2}\left(1+\frac{P_{mv}^{T}H_{mv}^{2}}{N_{mv}}\right)$$

where $P_{mv}^{T}$ is the transmission power of MV. $H_{mv}^{2}$ denotes the channel fading coefficient. $N_{mv}$ denotes the channel noise power, related to the link (V,B), and *W* is channel bandwidth.

### 3.3. Computation Model

The task m is executed in two ways: Local Computing and MEC-BS Computing. When many tasks are to be executed at the same time, the task must wait in the queue, including all pending tasks before the task m. Considering that the vehicle part follows the Poisson distribution, our modeling execution task m follows the M/ M/1 queue system [23], the probability of vehicle mission generation is $\lambda_{v}$.

#### 3.3.1. Local Computing

The main engine for local computing is the CPU on the VT, and the CPU performance is controlled by CPU cycle frequency ($f_{mv}$, CPU speed). It uses advanced DVFS technology to adjust the CPU speed by changing the cycle frequency of the CPU chip to achieve the minimum completion time and minimum power consumption of VT [13]. The computation execution time and energy consumption of MV by local computing is respectively given by:

(2) $$T_{mv}^{l}=\lambda_{v}L_{mv}f_{mv}^{-1}$$

(3) $$E_{mv}^{l}=\lambda_{v}kL_{mv}f_{mv}^{-1}$$

where *k* is the effective switched capacitance depending on the chip architecture. We set $k=10^{-11}$ [13].

**Definition** **1.** *Energy-Efficiency Cost (EEC): The weighted sum of energy consumption, computation completion time and availability of MEC-BS.*

When executed locally, there is no need to consider the availability of MEC-BS, so the EEC of MV in local computing is:

(4) $$Z_{mv}^{l}=\gamma_{mv}^{E}E_{mv}^{l}+\gamma_{mv}^{T}\left(T_{mv}^{l}-t_{max}\right)$$

where $0\leq \gamma_{mv}^{E},\gamma_{mv}^{T}\leq 1$, denote the weights of energy consumption and computation completion time to make them calculate on the same order of magnitude, and the VT makes different decisions. For example, a delay-sensitive application (such as online movies) might prefer to be big $\gamma_{mv}^{T}$, a device with low battery will choose a bigger $\gamma_{mv}^{E}$.

#### 3.3.2. MEC-BS Computing

Offloading process: The VT v offload the task m to the MEC-BS, and then the MEC-BS return the result to the VT v. It is worth noting that the availability of the MEC-BS is computed when the VT offloads the task to the MEC-BS, and there are three phases: (i) the transmitting phase; (ii) the MEC-BS execution phase; (iii) the result return phase.

In our algorithm, the availability of MEC-BS, $\eta_{mv}$ depended on the mobility problem and the admission control decision of MEC-BS [28]. The computation of the specific availability algorithm is given in Section V.

The time and energy consumption for the transmission phase is:

(5) $$T_{mv}^{ctrs}=\frac{\lambda_{v}D_{mv}}{R_{mv}\left(A\right)}$$

(6) $$E_{mv}^{ctrs}\left(A\right)=\lambda_{v}P_{mv}^{T}T_{mv}^{ctrs}\left(A\right)$$

The time and energy consumption for the execution phase is:

(7) $$T_{mv}^{cexe}=\lambda_{v}L_{mv}f_{c}^{-1}$$

(8) $$E_{mv}^{cexe}=\lambda_{v}kL_{mv}f_{c}^{2}$$

The penalty cost related to the availability of MEC-BS is:

(9) $$C_{mv}={\left(1-\eta_{mv}\right)}^{B}C_{pen}$$

where *B* is the number of MEC-BS, and $C_{pen}$ is a constant, ${\left(1-\eta_{mv}\right)}^{B}$ is the probability of offloading failure. The total completion time is the transmission time plus the execution time,

(10) $$T_{mv}^{c}=T_{mv}^{ctrs}+T_{mv}^{cexe}$$

The total energy consumption is the transmission energy plus the execution energy consumption,

(11) $$E_{mv}^{c}=E_{mv}^{ctrs}\left(A\right)+E_{mv}^{cexe}$$

According to Equations (6)–(12),the EEC of MV in MEC-BS is:

(12) $$Z_{mv}^{c}=\gamma_{mv}^{\alpha }C_{mv}+\gamma_{mv}^{E}E_{mv}^{c}+\gamma_{mv}^{T}\left(T_{mv}^{c}-t_{max}\right)$$

where $0\leq \gamma_{mv}^{\alpha }\leq 1$, is the weights of penalty cost.

### 3.4. Centralized Optimization Problem

The EEC of MV is:

(13) $$Z_{v}=\sum_{m=1}^{M}Z_{mv}=\sum_{m=1}^{M}\left(1-a_{mv}\right)Z_{mv}^{l}+a_{mv}Z_{mv}^{c}$$

To develop the optimal offloading decision A, we transforms the complex problem into EEC minimization problems under time and availability constraints. It is given by:

(14) $$\min_{A}\sum_{v=1}^{V}Z_{v}$$

$$\begin{array}{cc} \; & C1:\sum_{m=1}^{M}\left(1-a_{mv}\right)T_{mv}^{l}+a_{mv}T_{mv}^{c}\leq t_{max}\\ \; & C2:0\leq \eta_{mv}\leq 1\\ \; & C3:f_{c}\geq 0\\ \; & C4:f_{mv}\geq 0\\ \; & C5:a_{mv}\in 0,1\\ \; & C6:0\leq P_{mv}^{T}\leq P_{mv}^{max} \end{array}$$

C1 is completion time constraint. It specifies the total completion time that is required for the maximum completion time in all tasks of the Vehicle V application. (i.e., completion time deadline), $t_{max}$, which is set by the latency requirement for an application. C2 is availability constraints. Availability is between 0-1.C3 and C4 mean non-negative computing resources. C5 means the task m of the vehicle V is executed locally or offloaded to MEC-BS. C6 is the range of variation of the uplink transmission power. The above problem is a mixed integer programming problem. It is a non-convex and NP-hard problem [16].

## 4. Computation Offloading Game

We can adopt a game theory approach to achieve efficient offloading decisions for vehicles. Each vehicle is owned by a different individual. they pursue different interests, and finally achieve a solution that satisfies all parties. By studying the existence of Nash Equilibrium (NE) in potential games, we describes the above problem as a distributed potential game [12].

### 4.1. Game Formulation

The game model is denoted as $G=\{V,{\left(A_{v}\right)}_{v\in V},{\left(v_{v}\right)}_{v\in V}\}$, where $u_{v}$ is the cost function of VT v. The game model is specifically described as follows:

**Players:** Each VT is a participant. Participants compete for communication and computing resources, minimizing their communication and computing costs.

**Strategies:** Each VT takes energy consumption and time cost into account, with the aim of minimizing the total cost. It is worth noting that local calculations timeout will result in penalty costs. The offloading decision for VT v is $a_{v}\in A_{v}$.

**Payoffs:** The cost per vehicle v is the time cost and energy consumption in the communication and computation process. We denote $u_{v}\left(a_{v},a_{-v}\right)$ as the cost function, where $a_{-v}=\left(a_{1},\cdots ,a_{v-1},a_{v+1},\cdots ,a_{v}\right)$ is the offloading decision matrix consisting of all VT but VT v.

Each VT minimizes its own competitive cost:

(15) $$\min_{a_{v}\in 0,1}u_{v}\left(a_{v},a_{-v}\right)=\left(1-a_{v}\right)Z_{v}^{l}+a_{v}Z_{v}^{c},\forall v\in V$$

The proposed game model is solved using the existence of Nash Equilibrium (NE).

### 4.2. The Existence of NE

**Definition** **2.** A strategy profile $a^{*}=\left(a_{1}^{*},a_{2}^{*},\cdots ,a_{k}^{*}\right)$ is a NE of the game model.If at the equilibrium $a^{*}$, no player can further reduce its cost by unilaterally altering its strategy. i.e.,

(16) $$u_{v}\left(a_{v}^{*},a_{-v}^{*}\right)\leq u_{v}\left(a_{v},a_{-v}^{*}\right),\forall a_{v}\in 0,1,v\in V$$

NE has high self-stability. All vehicles will reach a balance when they get a solution that is satisfactory to each other, because each vehicle is selfish and acts for its own benefit. Then we study the existence of NE.

**Definition** **3.** *A game is called an exact potential game, if it admits a potential function ϕ(a) such that for every v∈V, and $a_{-v},a_{v}$, $a_{v}^{{}^{\prime }}\in A_{v}$, if*

(17) $$u_{v}\left(a_{v},a_{-v}\right)-u_{v}\left(a_{v}^{{}^{\prime }},a_{-v}\right)=\phi \left(a_{v},a_{-v}\right)-\phi \left(a_{v}^{{}^{\prime }},a_{-v}\right)$$

**Theorem** **1.** *Every ordinal potential game with finite strategy sets owns as least one pure-strategy NE and has the finite improvement property (FIP).*

The general potential game includes an exact potential game. A general potential game always admits a NE. Next, we prove that the game model is an exact potential game.

**Theorem** **2.** *The game model is an exact potential game with the potential function given in Equation (18), and hence, always has a NE and the FIP.*

(18) $$\begin{array}{cc} \phi (a)= & \left(1-a_{v}\right)\left\{\sum_{k\neq v}^{K}\left[\gamma_{k}^{E}\frac{P_{k}D_{k}}{R_{k}}+\gamma_{k}^{M}\left(T_{k}^{l}-t_{max}\right)\right]+Z_{v}^{l}\right\}\\ \; & +a_{v}\sum_{k=1}^{K}Z_{k}^{c} \end{array}$$

**Proof.**  Based on Equation (17), we have

(19) $$u_{v}\left(1,a_{-v}\right)-u_{v}\left(0,a_{-v}\right)=Z_{v}^{c}-Z_{v}^{l}$$

Based on Equation (19), $\phi (1,a_{-v})$ and $\phi (0,a_{-v})$ can be written as follows respectively, where 1 means the task offloaded to MEC-BS, and 0 means the task is executed locally,

(20) $$\begin{array}{cc} \phi (1,a_{-v}) & =\sum_{k=1}^{K}Z_{k}^{c}=Z_{v}^{c}+\sum_{k\neq v}^{K}Z_{k}^{c}\\ \; & =Z_{v}^{c}+\sum_{k\neq v}^{K}\left[\gamma_{k}^{E}\frac{P_{k}D_{k}}{R_{k}}+\gamma_{k}^{M}\left(T_{k}^{l}-t_{max}\right)\right] \end{array}$$

(21) $$\phi \left(0,a_{-v}\right)=\sum_{k\neq v}^{K}\left[\gamma_{k}^{E}\frac{P_{k}D_{k}}{R_{k}}+\gamma_{k}^{M}\left(T_{k}^{l}-t_{max}\right)\right]+Z_{v}^{l}$$

From Equations (20) and (21) we can achieve that:

(22) $$\phi \left(1,a_{-v}\right)-\phi \left(0,a_{-v}\right)=Z_{v}^{c}-Z_{v}^{l}$$

From Equations (19) and (22) we obtain that $\phi \left(1,a_{-v}\right)-\phi \left(0,a_{-v}\right)=u_{v}\left(1,a_{-v}\right)-u_{v}\left(0,a_{-v}\right)$ □

Therefore, The game model is an exact potential game. There is at least one NE and FIP.

### 4.3. Algorithm Description

In this section, we describe the game algorithm. Since the access node has complete information form VT and MEC-BS (offloading requests and responses, parameters of VTs and MEC-BS), it can compute the optimal offloading decision *A* and send *A* to all VTs. All VTs will follow the optimal offloading decision without deviation because of the property of NE. The NE derived by game algorithm denoted by Algorithm 1.
**Algorithm 1** Computation Offloading Decision for vehicle V**Require:** M:a sequence of M tasks of vehicle V $t_{max}$:maximum tolerance time $Iter_{max}$: maximum number of iterations**Ensure:**{A}:optimal offloading decision Initialization:$D_{mv}$,$L_{mv}$,$\gamma_{mv}^{E}$,$\gamma_{mv}^{T}$,$\gamma_{mv}^{a}$,$C_{pen}$ and iteration index t←1 **for**
m=1 to *M*
**do**  **while**
$t\leq Iter_{max}$
**do**   compute $R_{mv}$,$T_{mv}^{l}$,$E_{mv}^{l}$ by (1)–(3), respectively   compute $Z_{mv}^{l}=\gamma_{mv}^{E}E_{mv}^{l}+\gamma_{mv}^{T}\left(T_{mv}^{l}-t_{max}\right)$   compute $\eta_{mv}$ from Availability Prediction   compute $T_{mv}^{ctrs}$, $E_{mv}^{ctrs}$, $T_{mv}^{cexe}$,$E_{mv}^{cexe}$ by (6)–(8)(10), respectively   compute $T_{mv}^{c}=T_{mv}^{ctrs}+T_{mv}^{cexe}$, $E_{mv}^{c}=E_{mv}^{ctrs}+E_{mv}^{cexe}$, respectively   compute $Z_{mv}^{c}=\gamma_{mv}^{\alpha }\left(1-\eta_{mv}C_{pen}\right)+\gamma_{mv}^{E}E_{mv}^{c}+\gamma_{mv}^{T}\left(T_{mv}^{c}-t_{max}\right)$   **if**
$Z_{mv}^{c}\leq Z_{mv}^{l}$
**then**    $a_{mv}=1$   **else**    $a_{mv}=0$   **end if**   update $\eta_{mv}\left(t+1\right)=\eta_{mv}\left(t\right)$  **end while** **end for**

## 5. Vehicle Mobility Prediction: MEC-BS Availability Estimation

The computation model in Section 3 requires the MEC-BS availability $\eta_{mv}$. $\eta_{mv}$ depends on the mobility problem and the admission control decision of MEC-BS. Based on the collected data set, we decompose a time series prediction problem into several time series prediction problems based on EMD decomposition method, and then use LSTM method to separately predict, and finally use the sum of predicted sub data results as the output of the whole model.

### 5.1. MEC-BS Admission Control Based on Distance Priority

The MEC-BS may reject the offloading request. Therefore, we know that a very important factor affecting availability is the admission control of MEC-BS. We have adopted a distance-based access control strategy [27]. Specifically, the vehicles have different priorities from the MEC-BS in the same coverage area. The criteria for admission control policies may vary [29]. Other admission control strategies can also be used in this model. Before calculating the reception probability of MEC-BS availability, we introduce a concept similar to Signal Interference and Noise Ratio (SINR), which we call the External Service Ratio (ESR), for vehicle *v*, and other vehicles $v_{i},i\in 1,2\cdots$ within the same coverage. The ESR is:

(23) $$\beta_{ESR}\left(r\right)=\frac{gr^{-c}}{E_{s}+\sum g_{i}r_{i}^{-c}}$$

where *g* is the energy consumption (e.g., transmission power), we can assume that all $g_{i}\equiv g$, *r* is the distance from the vehicle v to the MEC-BS. We can estimate the distance *r* by the signal strength of the MEC-BS. *c* is a positive constant to indicate the sensitivity of the function, and $E_{s}$ indicates the energy consumption of the MEC-BS to handle its own work. From Equation (23), we can conclude that vehicles located closer to MEC-BS have relatively greater ESR. We assume that the MEC-BS has a preset constant $\hat{\beta }$. The MEC-BS is accepted only when the ESR of the vehicle offloading task is greater than the preset constant. (i.e., $\beta_{ESR}\geq \hat{\beta }$).

We define the virtual zone radius $R_{g}\left(r\right)$, which refers to the zone suppression of interference transmission around each receiver. Once an active vehicle appears in the virtual zone, the vehicle’s ESR is less than the preset ESR (i.e., $\beta_{ESR}\geq \hat{\beta }$).Any offloading request for vehicle v will be rejected [30].

The virtual zone radius $R_{g}\left(r\right)$ is given by:

(24) $$R_{g}\left(r\right)={\left(\frac{r_{-c}}{\hat{\beta }}-\frac{E_{s}}{g}\right)}^{-\frac{1}{c}}$$

Then the admission probability is given by:

(25) $$\zeta \left(r\right)=e^{-2\Pi \lambda_{au}{\left[R_{g}\left(r\right)\right]}^{2}}$$

where $\lambda_{au}$ is the density of active vehicles, because active vehicles are only part of the vehicle. We can denote $\lambda_{au}=c_{a}\lambda_{u}$, where $c_{a}$ is a constant. From Equations (24) and (25), we can see that the admission probability depends on the size of the virtual radius and the density of the active vehicle.

### 5.2. Mobility Problem: Estimated Dwell Time

**Figure 2:**
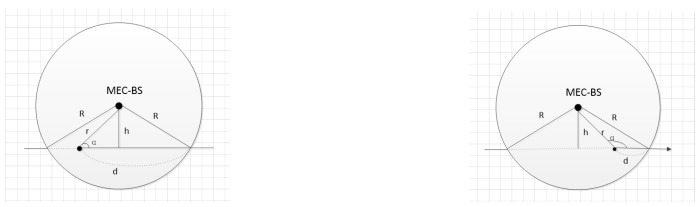
VT’s dwell trajectory in the MEC-BS coverage.

The VT’s dwell trajectory in the MEC-BS coverage is shown in Figure 2, where *R* is the coverage radius of the MEC-BS, *d* is the crossing distance within the coverage of the MEC-BS (0<d<2R), the variable α∈[0,Π] represents the angle between vehicle trajectories direction and MEC-BS, *r* represents the distance between the vehicle and the MEC-BS, and *h* represents the geometric height. The model assumes that the moving speed and direction of the vehicle do not change within the period of time. We can conclude that:

$$h=rsin\alpha ,0\leq \alpha \leq \Pi ,d=\sqrt{R^{2}-h^{2}}+rcos\alpha ,0\leq \alpha \leq \Pi$$

The dwell time *T* of the vehicle v in the coverage of the MEC-BS is given by:

(26) $$T\left(r\right)=\frac{d}{v_{0}}=\left(\frac{\sqrt{R^{2}-{\left(rsin\alpha \right)}^{2}}+rcos\alpha }{v_{0}}\right),0\leq \alpha \leq \Pi$$

so relative time is:

(27) $${\tilde{T}}_{t}=\frac{T-\tau }{T}$$

where *T* represents the predicted dwell time within the communication range, τ denotes the time required for the task to be completed.

### 5.3. Availability Prediction Based EMD and Deep Learning

#### 5.3.1. Data Preprocessing

The data of admission control and dwell time is converted into a machine learning processable format, such as qualitative variables need to be quantized, uniform size is not required in the same dimension, and so on. Integrate two sets of feature matrices to obtain a new feature matrix $I_{t}$ (28) through time correlation.

(28) $$I_{t}={\left[\zeta_{t},{\tilde{T}}_{t}\right]}^{H}$$

#### 5.3.2. EMD Decomposition

Time series decomposition techniques are widely used in time series related topics. EMD is chosen as a method for decomposing time series [31]. The formula is as follows:

(29) $$y\left(t\right)=\sum_{i=1}^{n}IMF_{i}\left(t\right)+r_{n}\left(t\right)$$

IMF (Intrinsic Mode Function) is a component of the original time series, and each IMF components contain local characteristic signals of different time scales of the original signal, $r_{n}$ is the residual component. There are different degrees of information entropy between these components, and we assume that they all have partial information components of the original time series. The total Information entropy $H=H_{1}\cup H_{2}\cup \cdots \cup H_{n}$, $H_{i}$ is the Infomation entropy of $IMF_{i}$. Therefore, it can be decomposed into several time series prediction problems.

**Figure 3:**
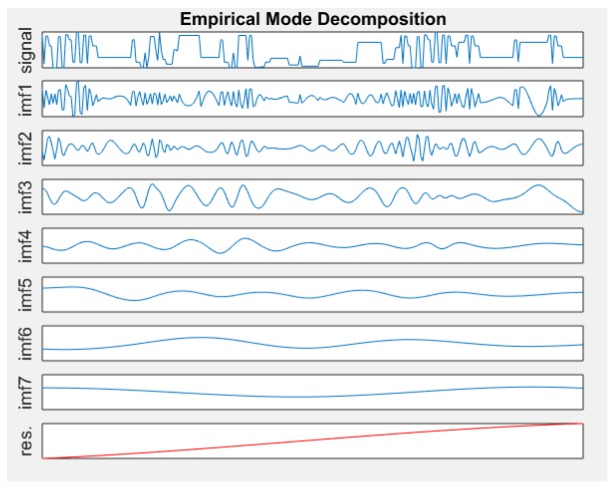
EMD Decomposition.

The Figure 3 shows that the original time series is decomposed into 7 sub-components, making it easier to build models and predictions.The decomposition data in each IMF component is used to construct a corresponding LSTM prediction model.

#### 5.3.3. LSTM Method for Prediction

**Figure 4:**
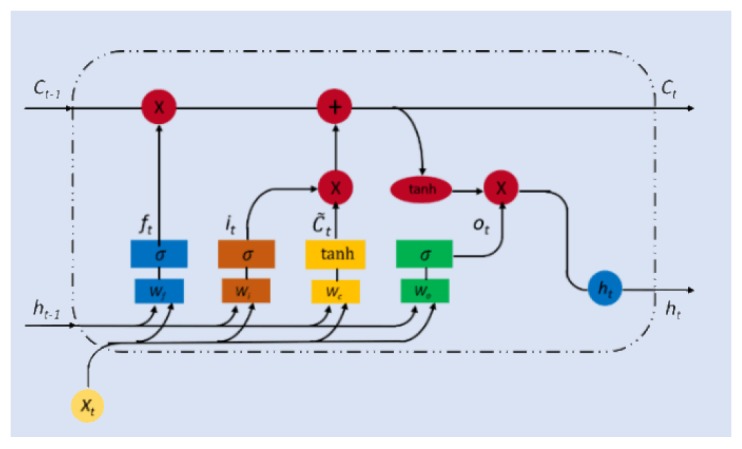
Architecture of LSTM (Red circle are arithmetic operators and the rectangles in different colours are the gates in LSTM).

A Long-short Term Memory(LSTM) method is proposed to predict the short-term availability due to its ability to handle long-term and short-term dependencies [32,33], LSTM shares similar architecture with Recurrent Neural Network(RNN), which are consists of one input layer, one hidden layer, and one output laye, which is shown in Figure 4. Typically, at each iteration t, the LSTM cell has the input layer $X_{t}$, a hidden layer and an output layer $h_{t}$. By adding a cell state, the LSTM cell is capable of handling long-term dependency of the sequence data, the previous output cell state, $C_{t-1}$ and current input cell state, ${\tilde{C}}_{t}$, both influence the current output cell state, $C_{t}$. Three gates control the information to flow into and out of the cell state which are the forget gate, the input gate, and the output gate, denoted as $f_{t}$,$i_{t}$, and $o_{t}$, respectively. The forget gate controls how much information from previous cell state should be forgotten by the current cell state. The input gate handles how much information from the current input layer flows into the current cell state. The output gate controls how much information from the current cell state would be conveyed into the current output layer. They can be calculated by the following equations,

(30) $$f_{t}=\sigma_{g}\left(W_{f}X_{t}+U_{f}h_{t-1}+b_{f}\right)$$

(31) $$i_{t}=\sigma_{g}\left(W_{i}X_{t}+U_{i}h_{t-1}+b_{i}\right)$$

(32) $$o_{t}=\sigma_{g}\left(W_{o}X_{t}+U_{o}h_{t-1}+b_{o}\right)$$

(33) $${\tilde{C}}_{t}=tanh\left(W_{c}X_{t}+U_{c}h_{t-1}+b_{c}\right)$$

where $W_{f}$, $W_{i}$, $W_{o}$, and $W_{c}$ are the weight matrices for mapping current input layer into three gates and current input cell state. $U_{f}$, $U_{i}$, $U_{o}$, and $U_{c}$ are the weight matrices for mapping the previous output layer into three gates and current input cell state. $b_{f}$, $b_{i}$, $b_{o}$, and $b_{c}$ are bias vectors for gate and input cell state calculation. $\sigma_{g}$ is the gate activation function which is normally a sigmoid function. tanh is the hyperbolic tangent function which is the activation function for current input cell state. Then, the current output cell state and output layer can be calculated by the following equations. Finally, the output of the LSTM prediction model should be the availability in the next time iteration.

(34) $$C_{t}=f_{t}*C_{t-1}+i_{t}*{\tilde{C}}_{t}$$

(35) $$h_{t}=o_{t}*tanh\left(C_{t}\right)$$

Finally, the input data $X_{t}$ is the data that each sub-component needs to predict. The predicted output through the LSTM method should be the sub-data at the next iteration, and the sum of the predicted sub-data results is taken as the output of the entire model.

EMD based LSTM method are as follows:

(1) Through time correlation, the admission probability and dwell time of feature matrices are integrated to obtain a new integrated feature matrix $I_{t}$.

(2) Transform the matrix into a form of 7 sub-data sets plus a redundancy through EMD decomposition.

(3) Training the sub-data to obtain the predicted results using LSTM method.( LSTM input: each sub-data by EMD; LSTM output: the predicted value of each sub-data.)

(4) Sum of each sub-predicted value as the total predicted output.

## 6. Performance Analysis

### 6.1. Experiment Profile

We consider the experimental scenario that V = 20 vehicles are traveling in one direction on a road with a distance of 5L. The density of the vehicles on the road is set as $\lambda_{v}=0.5$, and two MEC-BSs are set on 1/3, 2/3 of the road respectively. For wireless access, we set the bandwidth W = 5 MHz. We set the channel gain $H_{mv}=d_{vj}^{\varsigma }$ from vehicle v to MEC-BS. Where $d_{vj}$ is the distance from the vehicle to the MEC-BS, ς=4 is the path loss factor, we set the channel noise power $N_{mv}$ = 100 dBm, and initialize the weighting factor $\gamma_{mv}^{a}=0.4,\gamma_{mv}^{E}=\gamma_{mv}^{T}=0.3$.

### 6.2. Evaluation the LSTM Model

**Figure 5:**
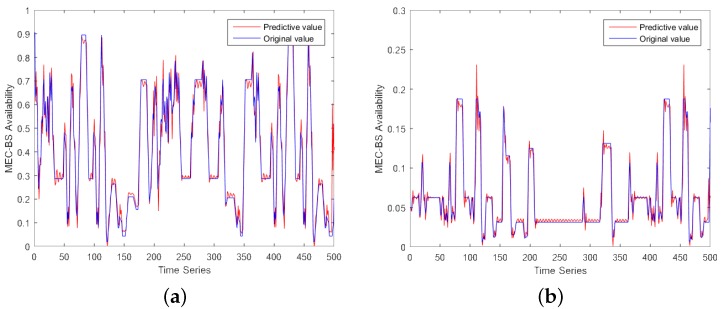
LSTM model predicts (**a**) IMF1 and (**b**) IMF4.

We used the LSTM model to predict moving VT in Section 5. In this section, From Figure 5a,b, we evaluated the LSTM model based on IMF1 and IMF4, respectively. In both figures, we use 500 data (the data used in this study were collected by Google cluster data-2011-2, which describes the offloading of tasks under a certain time constraint, which is very similar to the situation we studied), of which the first 495 are training sets and the last 5 are test data. We can see from the above figures that both curves are close to the actual data. In order to assess the accuracy of the simulation results, average absolute error(MAE) and root mean square relative error(RMSE) are chosen as evaluation indicators. The formulas are as (36) and (37).

(36) $$MAE=\frac{1}{n}\sum_{i=1}^{n}\left|r_{i}-{\hat{r}}_{i}\right|$$

(37) $$RMSE=\frac{1}{n}{\left[\sum_{i=1}^{n}{\left(r_{i}-{\hat{r}}_{i}\right)}^{2}\right]}^{\frac{1}{2}}$$

where *n* is the sample size, $r_{i}$ and ${\hat{r}}_{i}$ are real and predicted value, the LSTM model are compared with traditional predicting algorithms, including ARIMA (Autoregressive Integrated Moving Average Model), RT (Random Forest), KNN (K-Nearest Neighbor), GBDT (Gradient Boosting Decison Tree), the traditional prediction methods used for comparison are also tested under the same environment and data. The results are shown in the Table 2.

We can see that the MSE and RMSE of LSTM model are smaller, and they can evaluate the degree of data change. The smaller the value is, the better accuracy the prediction model has in describing experimental data, which indicating that the LSTM model based EMD method is very feasible to achieve the predicted effect. Because the LSTM has three gates that prevent gradient dispersion, it further illustrates the feasibility of using the LSTM method for prediction in a mobile environment.

### 6.3. Evaluation the Availability of MEC-BS

**Figure 6:**
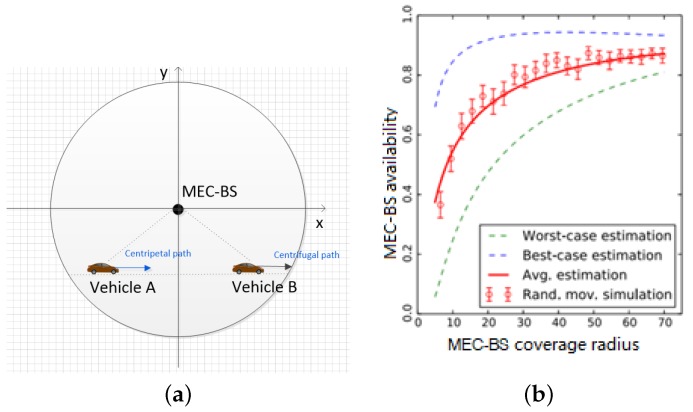
Mobility for (**a**) centripetal, centrifugal and random directions, (**b**) simulation results for centripetal (best-case), centrifugal (worst-case) and random direction movement.

In the third section of the computation model we need to use the availability of MEC-BS, then in the fifth section we specifically calculate the availability of MEC-BS. To verify the proposed motion guess, we simulated three modes of movement for the vehicle. As shown in Figure 6a, the vehicle has different options: centripetal, centrifugal, and random directions. The vehicle can be offloaded at any time during the MEC-BS coverage, and we record the probability of successful task offloading. For each movement mode, 4000 vehicle samples were independently tested and the MEC-BS coverage radius ranged from 5 to 70 m. The results of the analytical experiment are given in Figure 6b. The probability of successful offloading of the vehicle when the vehicle is moving centrifugally (i.e., the worst case) is much smaller than when it is moving centripetally (i.e., the best case). This is very likely because when performing centrifugation, the dwell time is relatively short, the dwell time is shorter, and the availability of MEC-BS is lower.

### 6.4. Impact of Weights

**Figure 7:**
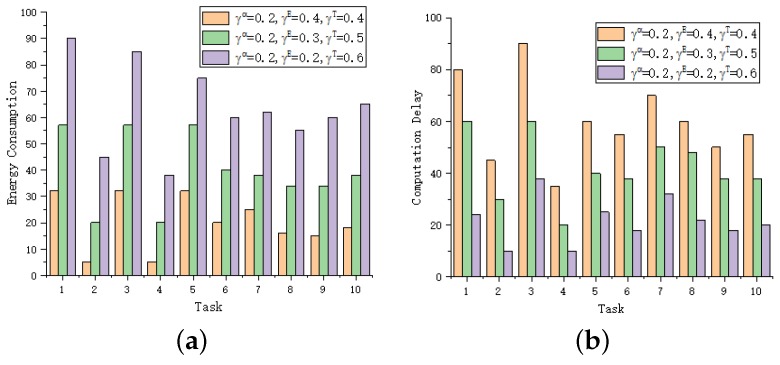
Comparison of energy consumption and computation time for different $\gamma_{mv}^{E}$, $\gamma_{mv}^{T}$. (**a**) Energy consumption. (**b**) Computation delay.

In this subsection, we examine the impact of weights, $\gamma_{mv}^{\alpha }$,$\gamma_{mv}^{E}$, $\gamma_{mv}^{T}$ on the penalty cost, the energy consumption and computation time of tasks with a different number of predecessors in Figure 7. To better observe the relationship between energy consumption and computation time, we set $\gamma_{mv}^{\alpha }$ = 0.2. For a given task, the energy consumption increase as the $\gamma_{mv}^{E}$ decreases, however, the changes of the computation time are opposite. This is reasonable since a large $\gamma_{mv}^{E}$ will lead to the increase of $P_{mv}^{T}$, which in turn causes the decrease of transmission power in edge execution.

### 6.5. Evaluation and comparison of the proposed algorithm

**Figure 8:**
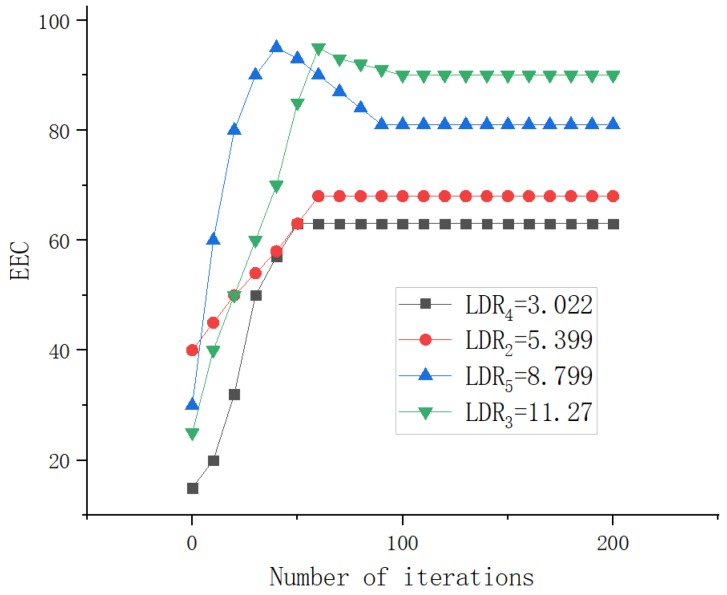
Dynamics of EEC of task with different LDRs.

First, we evaluate the impact of task complexity on EEC in Figure 8. We use the computing Load-input Data Ratio(LDR) to characterize the complexity of a task, i.e., $LDR=L_{mv}/D_{mv}$. We can observe that (i) the proposed algorithm can achieve converge within 100 iterations for all tasks; (ii) the task with larger LDR has higher EEC; (iii) the task with larger LDR has the lower speed of convergence, that is, the complexity of task can increase the convergence time. Therefore, reasonable LDR task setting plays an important role in improving offloading performance.

**Figure 9:**
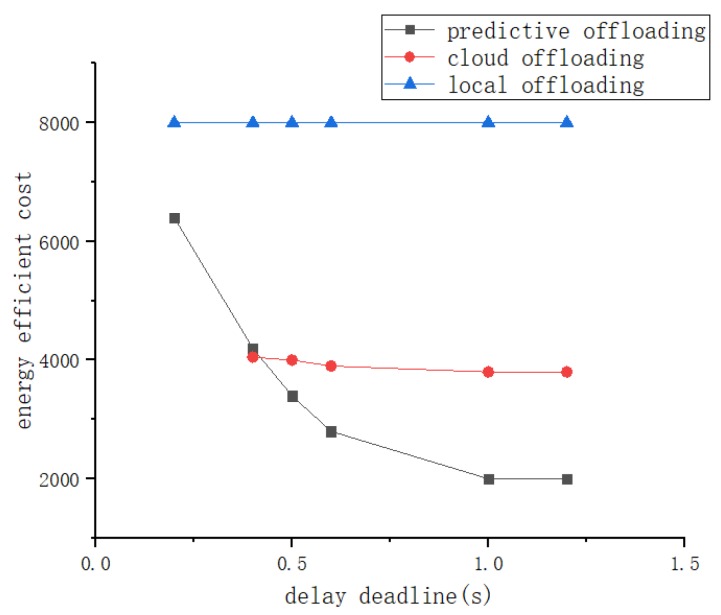
Comparison of EEC.

Under the strict deadline of completion time, the energy efficiency cost EEC is shown in Figure 9. In this section, in order to evaluate our algorithm. We compare it with the following algorithms: (1) local execution completely algorithm(LECA). All vehicles perform tasks locally; (2) cloud execution completely algorithm (CECA). All vehicle tasks are offloaded to MEC-BS.

We can draw some observations from Figure 9. First, our algorithm can reduce the EEC compared with LECA. When our algorithm remains stable, the cost can be reduced. This is because our algorithm can optimally choose to perform tasks locally or in MEC-BS according to the computational cost. Second, our algorithm has lower performance than CECA and LECA, because the proposed prediction model can save a lot of waiting time, the deadline of completion time becomes longer, offloading to the cloud becomes more flexible, and its EEC also decreases slightly.

**Figure 10:**
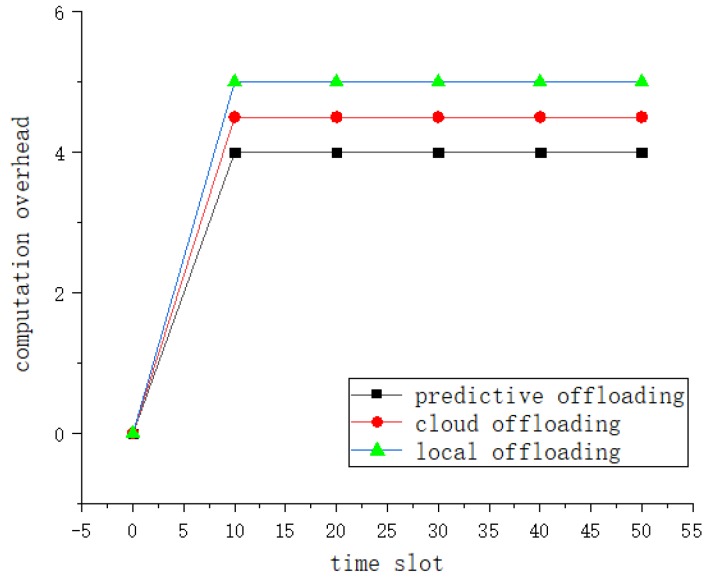
Comparison of energy consumption.

It shows the computational overhead of the VT dynamics in Figure 10. As time increases, the computational cost of all algorithms increases. At a certain time value, the computational overhead will not change. This is because after adding the MEC-BS availability prediction link, it is possible to study the load balancing of the MEC-BS and save the waiting time to some extent. so our proposed algorithm and LECA and CECA are less.

**Figure 11:**
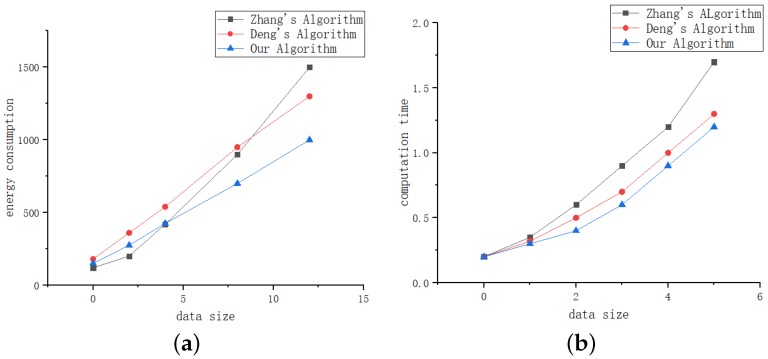
Comparison of energy consumption and application completion time for different algorithms. (**a**) Energy consumption. (**b**) Completion time.

In this part, we compare with the following algorithms: (i) the offloading game algorithm in [16] called Zhang’s algorithm; (ii) the resource scheduling algorithm in [18] called Deng’s algorithm. Figure 11 shows the completion time and energy consumption of the task at different input data sizes.

We can observe from Figure 11a that when the input data is small, Zhang’s algorithm consumes the least amount of energy. As the input data increases, the energy consumption grows faster because there is no corresponding energy control and adaptation mechanism in their algorithms, so our algorithm is more efficient when the input data is large. The Deng’s algorithm can adjust energy consumption according to resource scheduling. So when the input data is greater than 8, it is more energy efficient than Zhang’s algorithm, However, compared with these two algorithms. Our algorithm energy consumption has been low, which makes sense, our algorithm uses a predictive model which can save a large amount of waiting time. For the task of low transmission cost and high computational complexity, better computing resources are more important.

In addition, we can also observe from Figure 11b that our algorithm time increases more slowly as the input data gets larger. It can be observed that our algorithm is more effective when the input data is large, this is very recognized, when the input data is larger or can be understood as more tasks, because our algorithm has added LSTM prediction, can learn to continuously optimize the offloading results.

## 7. Conclusions

In this paper, we propose an LSTM-based availability prediction for optimized offloading in mobile edges. Firstly, considering the mobility of the vehicle, we propose a predictive availability scheme based on EMD and deep learning. Secondly, We jointly the availability of MEC, completion time, and energy consumption to minimize the overall cost. Then, we use the game method to get the optimal offloading decision. Finally, simulation experiments show that compared with the existing algorithms, the algorithm we proposed can effectively save energy and reduce the completion time.

For future work, we can consider parked vehicles and moving vehicles as part of the MEC, or combined with a variety of IOT devices for optimal offloading under different constraints, allowing full use of all resources, rational allocation of resources, and maximizing resource and time utilization.

## Figures and Tables

**Table 1:** Main Symbols And Their Meanings.

Symbols	Meanings
B	The number of MEC-BSs
V	The number of VT
M	The number of tasks
B	The set of MEC-BSs, B={0,1,⋯,B}
V	The set of VT, V={0,1,⋯,V}
M	The set of Tasks, M={0,1,⋯,M}
$L_{mv}$	Tasks load of m
$D_{mv}$	Input data size of m
$S_{mv}$	Received result size of m
$t_{max}$	Maximum tolerance time of m
*A*	Computation offloading decision
$P_{mv}^{T}$	Power of m in transmit
$P_{mv}^{T}$	Channel gain of m
$N_{mv}$	The power of the channel noise
*W*	Bandwidth channel
$R_{mv}$	Transmit rate of m
$\lambda_{v}$	The generation probability of m
$f_{mv}$	Local computational capability
$f_{c}$	MEC-BS computational capability
$\eta_{mv}$	MEC-BS availability
$T_{mv}^{l}/T_{mv}^{ctrs}/T_{mv}^{cexe}$	Local/transmission/MEC-BS time
$E_{mv}^{l}/E_{mv}^{ctrs}/E_{mv}^{cexe}$	Local/ transmission/MEC-BS energy consumption
$Z_{mv}^{l}/Z_{mv}^{l}$	Local/MEC-BS energy-efficiency cost
$\gamma_{mv}^{E}/\gamma_{mv}^{T}/\gamma_{mv}^{\alpha }$	The weight of energy consumption/time/penalty in offloading for m

**Table 2:** The prediction results of each model.

Models	MAE	RMSE
ARIMA	0.6624	0.8800
RT	0.5616	0.8002
KNN	0.5088	0.7251
GBDT	0.4363	0.7033
LSTM	0.4070	0.6678
